# Enzymatic Drivers of Cartilage Breakdown: Insights From a Bovine Osteoarthritis Explant Model

**DOI:** 10.1111/os.70202

**Published:** 2025-11-21

**Authors:** Austin Lawrence, Joseph Boesel, Katie Beier, Lucas Ratiani, Hayes Unrein, Ahmed Suparno Bahar Moni

**Affiliations:** ^1^ University of Toledo College of Medicine and Life Sciences Toledo Ohio USA; ^2^ Department of Orthopaedic Surgery University of Toledo College of Medicine and Life Sciences Toledo Ohio USA

**Keywords:** ADAMTS‐5, bovine explant model, cartilage degradation, COMP, MMP‐13, MMP‐9, osteoarthritis, TIMP‐3

## Abstract

**Objective:**

Osteoarthritis (OA) is a progressive joint disease characterized by cartilage degradation driven by matrix‐degrading enzymes. Reproducible ex vivo models are essential for studying early degenerative processes and evaluating potential therapeutics. However, there remains a lack of accessible, cost‐effective models that accurately replicate the biochemical environment and early‐stage damage of OA. This study aimed to develop and validate a bovine cartilage explant model that replicates key features of early OA through enzymatic induction of tissue damage.

**Methods:**

Bovine stifle cartilage explants were exposed to combinations of matrix metalloproteinases, aggrecanases, and cartilage biomarkers. Tissue damage was evaluated histologically, and semiquantitative scoring was used to assess structural changes. Statistical analyses were conducted to determine differences between treatment groups.

**Results:**

Enzyme‐treated samples exhibited significantly greater cartilage degradation compared to controls. The addition of cartilage oligomeric matrix protein (COMP) increased tissue damage, suggesting an active role in matrix destabilization. In contrast, the inclusion of TIMP‐3, a known protease inhibitor, did not reduce degradation, raising questions about its protective efficacy in this context.

**Conclusion:**

This chemically induced bovine model successfully simulates early cartilage degeneration consistent with OA pathology. Supported by recent literature on the roles of MMPs, ADAMTS‐5, and COMP in joint disease, the model offers a valuable platform for future studies on OA mechanisms and therapeutic screening.

## Graphical abstract

A bovine cartilage explant model was developed to simulate early osteoarthritis using matrix‐degrading enzymes. Histological analysis revealed synergistic cartilage damage from MMPs, ADAMTS‐5, and COMP. This ex vivo model offers a reproducible platform to study OA mechanisms and test therapeutic interventions.

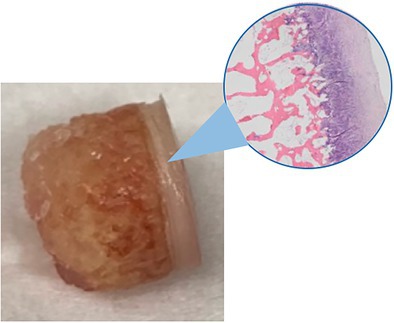

## 1 Introduction

Osteoarthritis (OA) is a degenerative joint disease affecting more than 300 million individuals worldwide [1]. Destruction of joint articular cartilage (AC) is the striking feature of OA, resulting in pain, stiffness, inflammation, and loss of mobility [2]. Due to the slowly progressive nature, OA is difficult to detect early, leaving limited treatment options available for patients once symptoms are more pronounced [3]. Hence, recent research has focused on detecting biomarkers associated with OA. Several inflammatory enzymes, including various matrix metalloproteinases (MMPs) and metalloproteinases with thrombospondin type I motifs (ADAMTSs), as well as the cartilage oligomeric matrix protein (COMP), have been investigated in relation to the pathogenesis of OA [4, 5]. Since these proteins are directly upregulated in OA, this study aims to develop a bovine model for chemically induced OA using the proteins.

The MMPs involved in remodeling the extracellular matrix of cartilage have been strongly associated with the pathogenesis of OA [6]. Specifically, MMPs‐1, 2, 9, and 13 have been upregulated in synovial fluid, leading to cartilage destruction [6, 7]. Aggrecan, one of the proteoglycans that make up cartilage, protects against collagen degradation [8]. This can be cleaved by the ADAMTS family of enzymes, including ADAMTS‐5, which can be overactive and cause degenerative joint damage [9]. Therefore, exposure to a combination of these enzymes will theoretically produce the characteristic damage seen in OA.

The tissue inhibitor of metalloproteinase 3 (TIMP‐3) serves as a regulator protein for the MMPs, inactivating these metalloproteinases during inflammatory processes [10]. In addition to inhibiting the MMPs, TIMP‐3 has also been shown to inhibit the activity of tumor necrosis factor alpha (TNF‐α) converting enzyme [11, 12]. These combined protective roles of TIMP‐3 can be further investigated by exposing AC to both MMPs and TIMP‐3.

Though COMP concentrations have been elevated in the synovial fluid of individuals with OA, the exact function of the protein remains unclear [13]. COMP is a member of the thrombospondin family of proteins and is found in AC, tendons, and synovial tissue. The protein is known to be a contributor to the matrix assembly of AC; however, whether COMP is simply a degradation product or both a product and contributor to the pathogenesis of OA has yet to be discovered [14].

Animal models allow for an inexpensive and efficient way of researching diseases. The bovine stifle joint is anatomically similar to the human knee, one of the weight‐bearing joints that commonly develops OA. While previous OA models have used mechanical or surgical induction, few have examined the combined effects of multiple matrix‐degrading enzymes and cartilage biomarkers in a controlled ex vivo setting. This study addresses a critical gap by chemically replicating early OA‐related cartilage degradation using enzymes at physiologically relevant concentrations. Therefore, in this study, we exposed bovine stifle joints to MMP‐9, MMP‐13, ADAMTS‐5, and COMP to develop a model for OA. TIMP‐3 was also used to investigate its proposed inhibitory effects on the MMP enzymes. The purposes of this study were to (i) establish a reproducible ex vivo OA model by exposing bovine cartilage plugs to a combination of enzymes known to be upregulated in human OA, (ii) investigate the role of COMP in matrix destabilization, evaluating whether it acts as more than a biomarker by contributing to cartilage breakdown, and (iii) assess the protective capacity of TIMP‐3 in the presence of matrix‐degrading enzymes to explore its therapeutic potential. Results from the study will provide a model for future research regarding potential treatment options as well as improve the current understanding of the disease.

## 2 Methods

### 2.1 Sample Collection and Preparation

**FIGURE 1:**
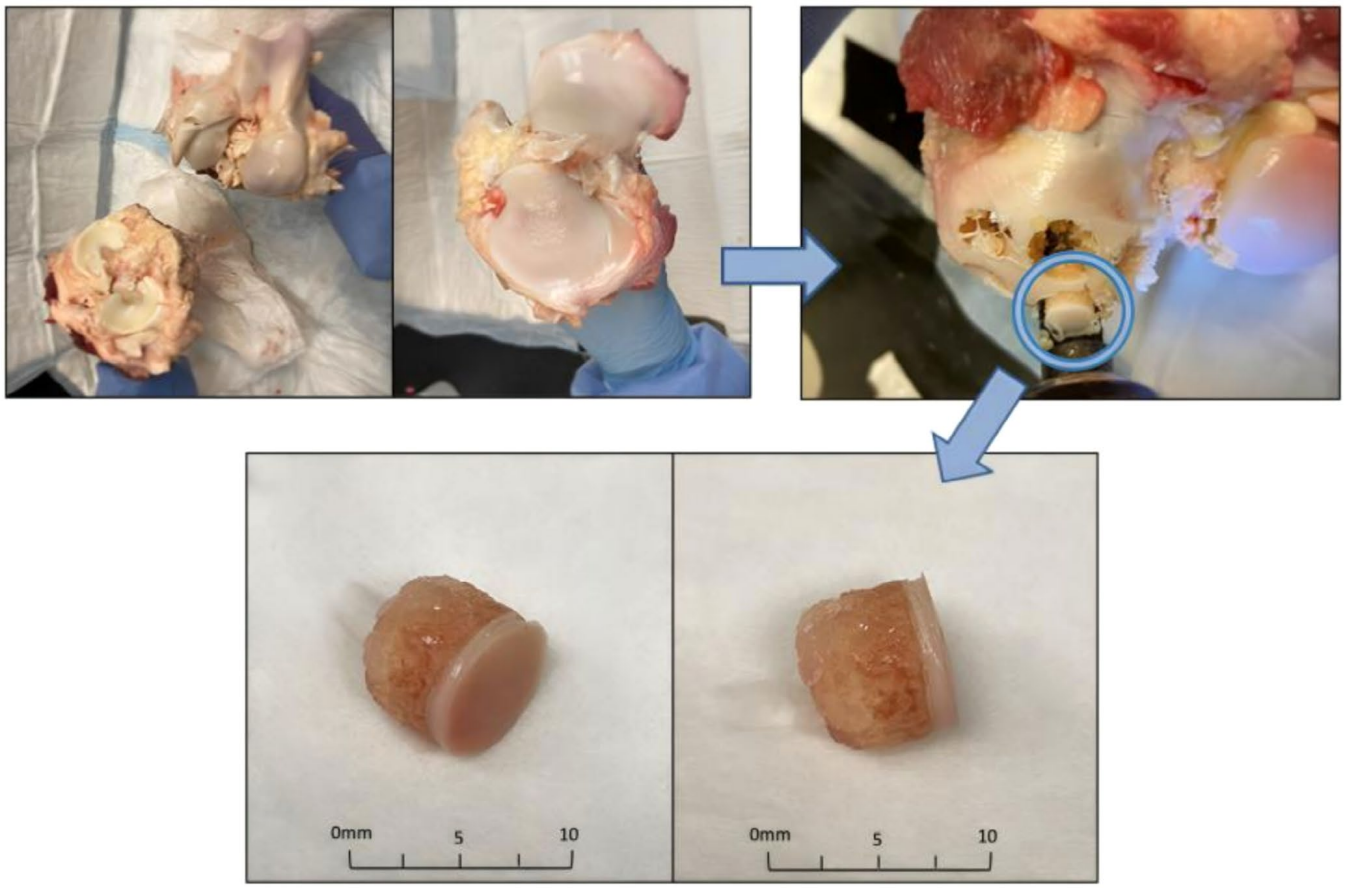
Cartilage plugs (5 mm in height) harvested from 48 h postmortem bovine stifle joints.

**FIGURE 2:**
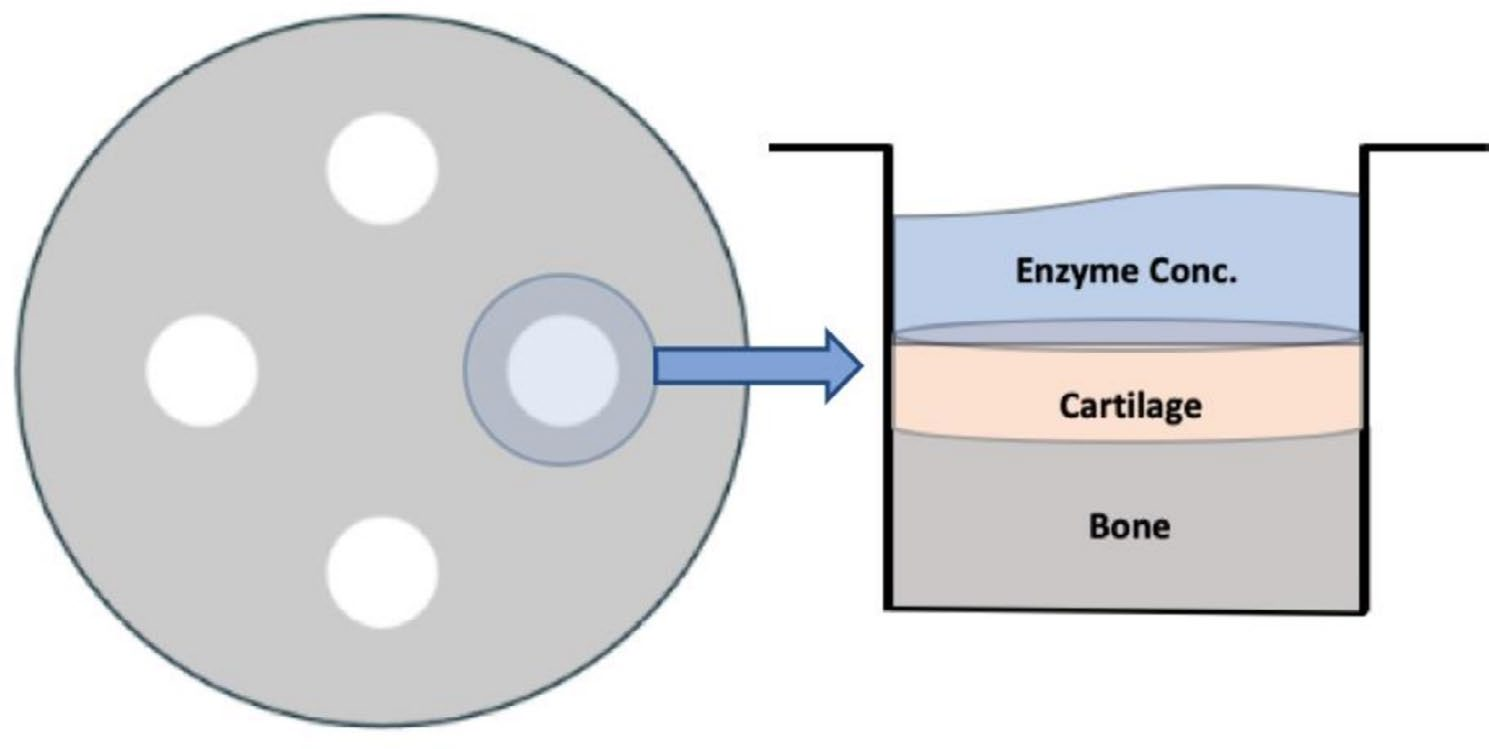
Well plate setup for cartilage plugs. Each plate will contain the same group (i.e., all 4 cartilage plugs on this plate will be exposed to Group A conditions).

Bovine stifle joints were sourced from healthy, juvenile cows (age: 1–3 years) for the development of an OA model. The bovine stifle joint was selected due to its anatomical similarity to the human knee, which is a primary weight‐bearing joint commonly affected by OA. Using a surgical power drill, cylindrical cartilage plugs measuring 8 mm in diameter and 3 mm in thickness were extracted (Figure 1). The cartilage plugs were then transferred to sterile agarose plates, where they were gently embedded in a solidified 2% agarose gel to keep them secure during enzyme treatment (Figure 2). The agarose gel was prepared by dissolving 50 g of agarose in 2500 mL of 1 × TAE buffer, autoclaving, and then solidifying the agarose in culture plates.

### 2.2 Enzyme Treatment

The bovine cartilage plugs were exposed to a combination of recombinant enzymes, including matrix metalloproteinases (MMP‐9 and MMP‐13), aggrecanase ADAMTS‐5, COMP, and tissue inhibitor of metalloproteinases‐3 (TIMP‐3) to induce OA‐like degradation (Table 1). MMP‐9, MMP‐13, and ADAMTS‐5 are involved in cartilage breakdown, while COMP is a structural protein upregulated in OA. TIMP‐3, a natural inhibitor of MMPs, was included to evaluate its potential role in modulating matrix degradation. TIMP‐3 is typically downregulated in OA, leading to increased activity of MMPs and ADAMTSs, which accelerate cartilage destruction. To initiate the enzymatic degradation, each group of cartilage plugs was exposed to a specific combination of these enzymes. The specific concentrations of enzymes were chosen based on previous studies on enzyme concentrations found in human and animal synovial fluid of those with symptomatic knee OA. The total volume in each well was 200 μL (Table 2).

**TABLE 1:** Enzyme source and catalog information.

Enzyme	Catalog no.	Source
5 μg Matrix Metalloproteinase‐9/Gelatinase B	PF024	Sigma‐Aldrich, Saint Louis, MO, USA
5 μg Matrix Metalloproteinase‐13/Collagenase 3	ab227435	Abcam, Cambridge, UK
5 μg ADAMTS‐5/Aggrecanase	CC1034	Sigma‐Aldrich, Saint Louis, MO, USA
100 μg Cartilage oligomeric matrix protein (COMP)/Thrombospondin‐5	ab174082	Abcam, Cambridge, UK
10 μg Tissue Inhibitor of Metalloproteinase‐3 (TIMP‐3)	T1327	Sigma‐Aldrich, Saint Louis, MO, USA

**TABLE 2:** Outline for how much enzyme to place in each well. The control is just 200 μL of PBS on top of the cartilage plug (Ex: Place 40 μL of MMP‐9, 40 μL of MMP‐13, 40 μL of ADAMTS‐5, and 80 μL of PBS into each of the 4 wells in the plate. Each well should have a total of 200 μL in it).

Add	40 μL	40 μL	40 μL	40 μL	40 μL
Group A	MMP‐9	MMP‐13	ADAMTS‐5	PBS	PBS
Group B	MMP‐9	MMP‐13	ADAMTS‐5	COMP	PBS
Group C	MMP‐9	MMP‐13	ADAMTS‐5	COMP	TIMP‐3
Control D	PBS	PBS	PBS	PBS	PBS

In Group A, the joints were exposed to 30 ng/mL MMP‐9, 15 ng/mL MMP‐13, and 15 ng/mL ADAMTS‐5, along with 80 μL of PBS. This combination of enzymes was selected to promote cartilage breakdown, simulating OA progression.

Group B received 30 ng/mL MMP‐9, 15 ng/mL MMP‐13, 15 ng/mL ADAMTS‐5, and 5000 ng/mL COMP, with 40 μL of PBS. The addition of COMP was hypothesized to increase cartilage degradation, and this was expected to be more apparent in histological analysis compared to Group A.

In Group C, the treatment included 30 ng/mL MMP‐9, 15 ng/mL MMP‐13, 15 ng/mL ADAMTS‐5, 5000 ng/mL COMP, and 200 ng/mL TIMP‐3. This group was designed to model a scenario where OA progression is mitigated by the inhibitory effects of TIMP‐3. It was expected that TIMP‐3 would reduce the activity of MMPs and ADAMTS, resulting in slower cartilage degradation compared to Groups A and B, potentially providing a therapeutic avenue for mitigating OA damage.

Group D, the control group, was treated with PBS only. As this group did not receive any enzymes, it was expected to show no significant cartilage degradation, providing a baseline for comparison with the other treatment groups. The concentrations of the enzymes were selected based on findings from prior studies, which suggested that these levels would effectively induce cartilage breakdown in the bovine model.

The plates were then incubated at 37°C with 5% CO_2_ for 44 h, with gentle agitation every 10–12 h. After the incubation period, the samples were removed, washed three times with PBS, and prepared for histology analysis.

### 2.3 Histological Analysis

After the PBS wash cycle, the AC samples were harvested and fixed in 4% paraformaldehyde for 48 h. The samples were then decalcified in EDTA solution and embedded in paraffin. Sections (5 μm thick) were cut from each sample using a microtome, mounted on glass slides, and stained with Hematoxylin and eosin (H&E), periodic acid‐Schiff (PAS), and Masson's Trichrome. The PAS stain was used to visualize glycosaminoglycans (GAGs) in the extracellular matrix, highlighting cartilage integrity and composition. H&E staining was used to assess general tissue morphology, revealing features like cellularity and inflammation. Masson's Trichrome stain was employed to detect collagen fibers in the extracellular matrix, providing insights into collagen integrity and loss. Histological grading was performed using a scoring system from 0 (no damage) to 3 (severe damage), assessing features such as cartilage surface integrity, cell density, and matrix degradation.

### 2.4 Statistical Analysis

Histological scores were analyzed to compare the effects of the different enzyme treatments versus control samples. RStudio, Version 2023.12.1 + 402, Posit Software, PBC, Boston, MA, USA, was utilized with a *p* < 0.05 level of significance. For the histological analysis, the normality of the data was first assessed using the Kolmogorov–Smirnov test. Subsequently, a Kruskal‐Wallis test was performed to determine whether there were significant differences between the enzyme treatment groups. Post hoc analyses were conducted using Dunn's test and Cliff's Delta test to evaluate the magnitude of differences between groups and assess the statistical significance of the findings.

## 3 Results

A total of 47 cartilage plugs were initially harvested from the femoral and tibial stifle cartilage of four bovine stifle joints, each obtained from a different cow. Of these, 15 plugs were excluded due to poor cartilage composition upon initial inspection. Specifically, 32 plugs were allocated into four experimental enzyme groups (Group A: MMPs + ADAMTS, Group B: MMPs + ADAMTS + COMP, Group C: MMPs + ADAMTS + COMP + TIMP, and a negative control treated with PBS) and subjected to enzyme degradation.

### 3.1 Histology Analysis

Histological analysis was performed on 32 plugs distributed among the four enzyme groups, with damage grades recorded for H&E, Masson's Trichrome, and PAS staining methods. Each stain revealed characteristic differences in cartilage damage between the experimental groups.

**FIGURE 3:**
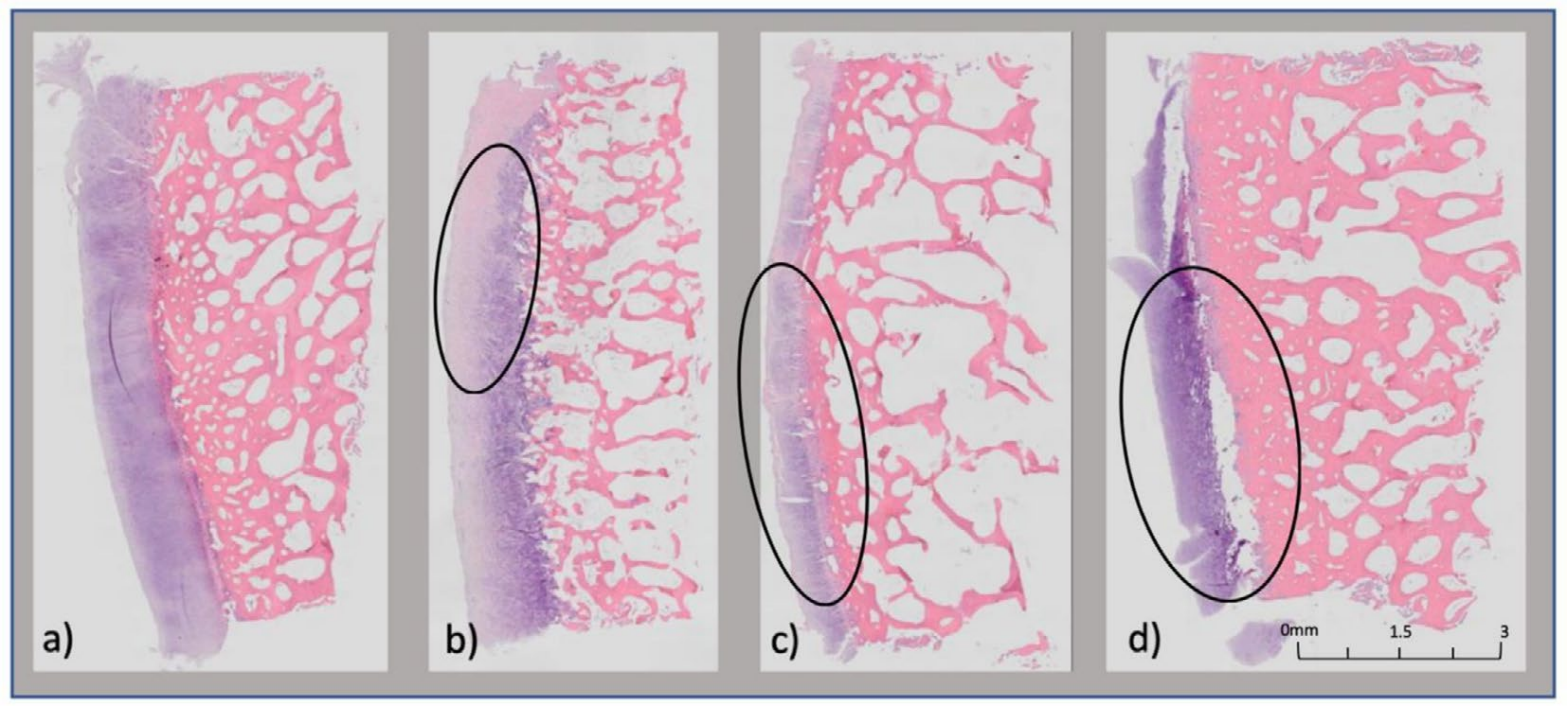
(a–d): H&E histological tissue damage for each group. (a) No damage noted for the control group. (b) Mild damage noted for Group A (black). (c) Moderate damage noted for Group B (black). (d) Severe damage noted for Group C (black).

Damage grades assessed via H&E staining demonstrated clear differences in cartilage integrity across the groups. The PBS (negative control) consistently showed no damage (grade 0) across all plugs, reflecting intact cartilage structure (Figure 3a). In contrast, the MMPs + ADAMTS group exhibited mild to moderate damage (grades 1–2, Figure 3b), while the MMPs + ADAMTS + COMP group ranged from mild to severe damage (grades 1–3, Figure 3c). Interestingly, the TIMP3‐treated group showed moderate to severe damage (grades 2–3), displaying no protective effect and arguably increased damage compared to other experimental groups (Figure 3d).

**FIGURE 4:**
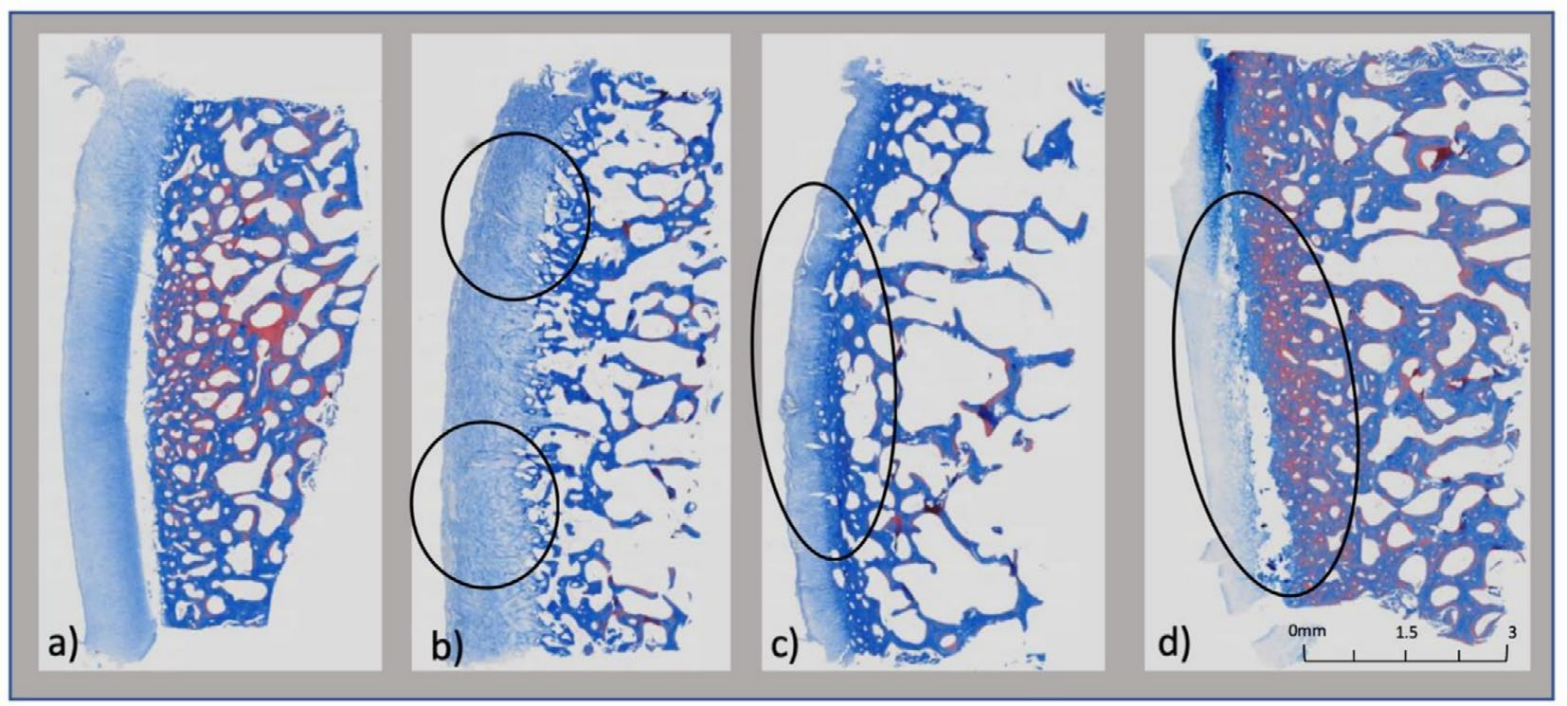
(a–d): Trichrome histological collagen degradation for each group. (a) No degradation noted for the control group. (b) Mild degradation noted for Group A (black). (c) Moderate degradation noted for Group B (black). (d) Mild–moderate degradation noted for Group C (black).

Masson's Trichrome staining highlighted collagen degradation in the cartilage matrix. Similar to H&E, the PBS group displayed no detectable damage (grade 0), confirming the absence of matrix degradation in the control (Figure 4a). The MMPs + ADAMTS group showed mild to moderate collagen damage (grades 1–2, Figure 4b), whereas the MMPs + ADAMTS + COMP group exhibited mild to severe damage (grades 1–3, Figure 4c). Interestingly, the TIMP3‐treated group demonstrated a mixed pattern, with damage grades ranging from 1 to 3, suggesting little to no inhibition of enzymatic activity (Figure 4d).

**FIGURE 5:**
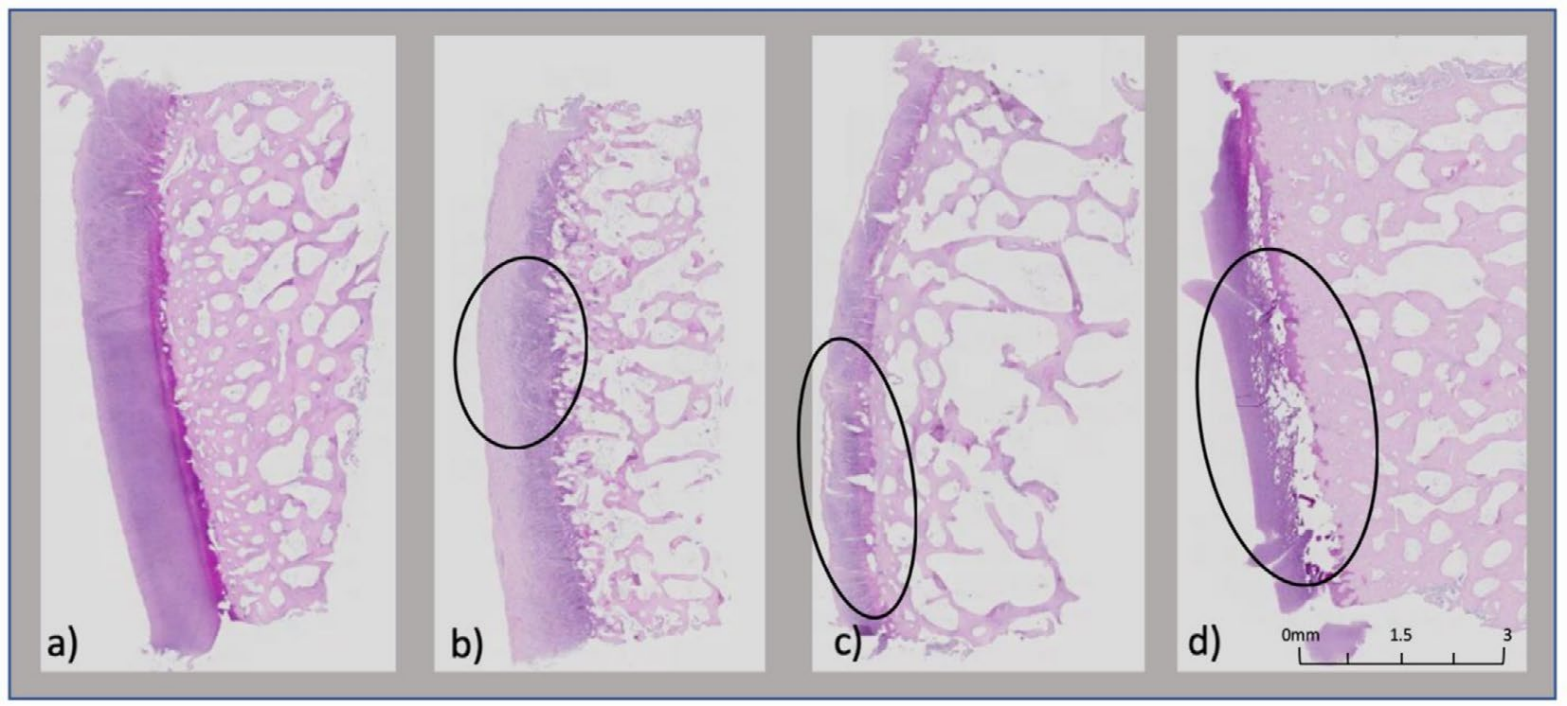
(a–d): PAS histological GAG depletion for each group. (a) No depletion noted for the control group. (b) Mild depletion noted for Group A (black). (c) Moderate depletion noted for Group B (black). (d) Moderate–severe damage noted for Group C (black).

PAS staining assessed GAG depletion in the cartilage matrix. The PBS control group consistently showed no GAG depletion (grade 0), reflecting normal cartilage composition (Figure 5a). In the MMPs + ADAMTS group, depletion grades ranged from 1 to 2, indicating moderate GAG loss (Figure 5b). The MMPs + ADAMTS + COMP group exhibited a broader range of depletion, with grades from 1 to 3 (Figure 5c). The TIMP3‐treated group showed similar variability (grades 1–3), yet favored increased GAG depletion compared to the other groups (Figure 5d).

### 3.2 Statistical Analysis

**FIGURE 6:**
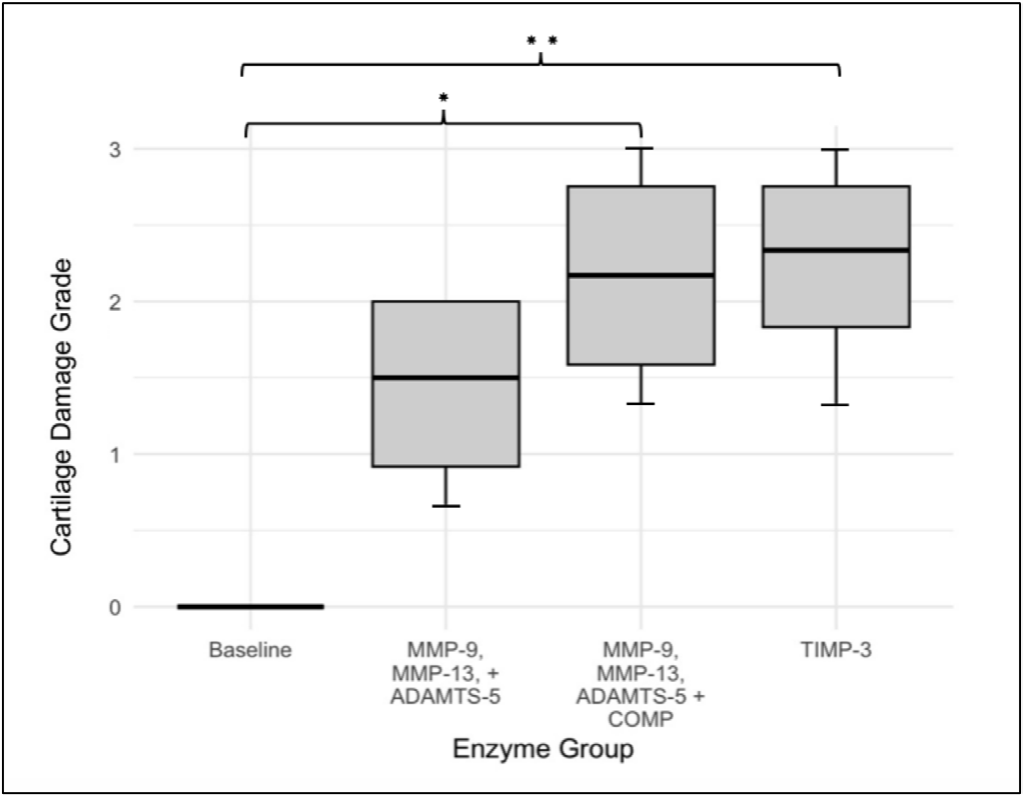
Distribution of histological scores based on tissue damage among the four groups.

The Kruskal‐Wallis test was used to compare the distribution of cartilage damage grades across the four experimental groups. The results showed a significant difference between the groups (chi‐squared = 10.085, *p*‐value = 0.01786), indicating that enzyme treatment groups differed in terms of cartilage damage severity. Dunn's post hoc test was conducted to identify specific group differences. The results revealed that plugs from Group 3 and Group 4 [MMPs + ADAMTS‐5 + COMP and TIMP‐3‐treated] have significantly greater damage compared to Group 1 (PBS control), with *p*‐values of 0.0228 and 0.0144, respectively. This suggests that these enzyme treatments resulted in significantly greater cartilage degradation compared to healthy baseline cartilage. The box plot shown in Figure 6 further illustrates these findings, highlighting the distribution of cartilage damage grades across the experimental groups.

The Cliff's Delta values indicated large effect sizes between the PBS control and enzyme‐treated groups. Specifically, for comparisons between the PBS control and MMPs + ADAMTS + COMP and TIMP3 groups, values of −1 (large) reflected substantial differences in cartilage damage.

## 4 Discussion

### 4.1 Main Findings

This study successfully developed a reproducible bovine ex vivo model of OA through enzymatic degradation of cartilage tissue. Histological staining and semiquantitative grading confirmed that treatment with MMP‐9, MMP‐13, ADAMTS‐5, and COMP induced characteristic structural deterioration similar to early‐stage OA. Notably, the addition of COMP exacerbated tissue damage, and the inclusion of TIMP‐3 did not provide the expected protective effects, challenging current assumptions about its role in cartilage preservation.

### 4.2 Interpretation of Results

Matrix metalloproteinases and aggrecanases play a central role in cartilage matrix breakdown. Our findings align with prior research showing that MMP‐13 is a key collagenase in OA and that ADAMTS‐5 is the primary aggrecan‐degrading enzyme [4, 7, 9]. Consistent with previous studies [15, 16, 17], the combined presence of MMPs and ADAMTS‐5 induced more pronounced degradation than either enzyme alone.

A novel insight from this study is the damaging role of exogenous COMP. Traditionally viewed as a structural matrix protein and OA biomarker [5, 13], COMP appeared to exacerbate cartilage breakdown when present alongside MMPs and ADAMTS‐5. This suggests that COMP may function as an active contributor to matrix destabilization in OA pathogenesis [18], rather than serving as a passive marker of disease.

In contrast, TIMP‐3—a known inhibitor of MMPs and ADAMTSs—did not mitigate matrix damage in this model. This contradicts its presumed therapeutic potential and may reflect one or more limitations: insufficient concentration, in vitro instability, or differences between soluble versus matrix‐bound TIMP‐3 activity [12, 19]. These findings raise questions about how TIMP‐3 behaves in different biological contexts and suggest that further studies should account for localization and protease activation dynamics.

Our results also support the growing hypothesis that OA pathogenesis is multifactorial and not solely attributable to enzymatic cartilage breakdown. The potential for COMP to serve as a disease effector adds a new dimension to its role in OA, particularly given its correlation with symptom severity and progression in clinical studies [3, 13].

### 4.3 Limitations

While the present study effectively demonstrated enzyme‐induced cartilage degradation, several areas could enhance the model's applicability in future work. For example, the absence of mechanical loading limits the ability to replicate joint biomechanics, which are known to influence cartilage health and degradation. Incorporating dynamic stress conditions could provide a more physiologically accurate environment. Additionally, only a single concentration of TIMP‐3 was evaluated; future studies exploring a range of concentrations could better characterize its inhibitory capacity and therapeutic window. Lastly, the 44‐h experimental window may not fully capture the time‐dependent aspects of matrix remodeling and enzyme activity. Extending the incubation period could reveal longer‐term effects and better simulate chronic disease progression. With the integration of mechanical stimulation, dose–response studies, and extended observation periods, this explant system holds strong potential for advancing OA research and preclinical therapeutic testing.

### 4.4 Strengths

One of the model's key strengths lies in its use of bovine stifle cartilage, which closely mirrors the structural and biomechanical characteristics of the human knee—making the findings more translationally applicable than smaller animal models. Additionally, the model allows precise control over the molecular environment, enabling the systematic dissection of individual and combined effects of disease‐associated proteins such as MMPs, ADAMTS‐5, COMP, and TIMP‐3. Unlike surgical or mechanically induced OA models, this system isolates biochemical drivers of cartilage degradation, allowing researchers to evaluate the direct effects of therapeutic inhibitors and candidate biomarkers in a highly reproducible and cost‐effective format. Its scalability and compatibility with high‐resolution histological analysis further position it as a valuable platform for both mechanistic studies and preclinical drug screening in OA research.

## 5 Conclusion

This study presents a reproducible ex vivo bovine model for OA that effectively simulates early enzymatic cartilage degeneration using a cocktail of matrix‐degrading enzymes. By mimicking the proteolytic environment observed in human OA using MMP‐9, MMP‐13, ADAMTS‐5, and COMP, this model offers a controlled and scalable system for studying cartilage breakdown. Notably, the lack of protective effect from TIMP‐3 underscores the complexity of protease regulation and highlights potential limitations of endogenous inhibitors in therapeutic applications.

The bovine model provides a valuable platform for the controlled study of early degenerative changes and the screening of therapeutic candidates targeting protease activity. It also enables exploration of disease‐modifying interventions, such as small‐molecule inhibitors of ADAMTS‐5, monoclonal antibodies, or gene‐based therapies. Future work should explore dose–response relationships, longer incubation timelines, and integration of biomechanical testing to expand the translational relevance of the model. Incorporating systemic inflammatory factors may further elucidate the molecular dynamics of OA progression. Ultimately, this work supports the use of bovine explants as a cost‐effective and biologically relevant OA model system, advancing our ability to study cartilage degeneration and test disease‐modifying OA drugs.

## Author Contributions

**Austin Lawrence:** conceptualization, investigation, writing – original draft, methodology, validation, visualization, writing – review and editing, software, formal analysis, project administration, data curation, supervision, resources. **Joseph Boesel:** conceptualization, investigation, writing – original draft, methodology, data curation, supervision, writing – review and editing. **Katie Beier:** conceptualization, investigation, methodology, data curation. **Lucas Ratiani:** conceptualization, investigation, methodology, data curation. **Hayes Unrein:** conceptualization, investigation, methodology, data curation. **Ahmed Suparno Bahar Moni:** conceptualization, funding acquisition, project administration, methodology, writing – review and editing, resources.

## Disclosure

All authors listed meet the authorship criteria according to the latest guidelines.of the International Committee of Medical Journal Editors. All authors are in agreement with the manuscript.

## Conflicts of Interest

The authors declare no conflicts of interest.
